# Neuronal Correlates of Maladaptive Coping: An EEG-Study in Tinnitus Patients

**DOI:** 10.1371/journal.pone.0088253

**Published:** 2014-02-18

**Authors:** Sven Vanneste, Kathleen Joos, Berthold Langguth, Wing Ting To, Dirk De Ridder

**Affiliations:** 1 Department of Translational Neuroscience, Faculty of Medicine, University of Antwerp, Antwerp, Belgium; 2 School of Behavioral and Brain Sciences, The University of Texas at Dallas, Richardson, Texas, United States of America; 3 Department of Psychiatry and Psychotherapy, University Regensburg, Regensburg, Germany; 4 Faculty of Social Work and Welfare Studies, University College Ghent, Ghent, Belgium; 5 Department of Surgical Sciences, Dunedin School of Medicine, University of Otago, Dunedin, New Zealand; 6 BRAI^2^N, Sint Augustinus Hospital, Antwerp, Belgium; University of Salamanca- Institute for Neuroscience of Castille and Leon and Medical School, Spain

## Abstract

Here we aimed to investigate the neuronal correlates of different coping styles in patients suffering from chronic tinnitus. Adaptive and maladaptive coping styles were determined in 85 tinnitus patients. Based on resting state EEG recordings, coping related differences in brain activity and connectivity were found. Maladaptive coping behavior was related to increases in subjective tinnitus loudness and distress, higher tinnitus severity and higher depression scores. EEG recordings demonstrated increased alpha activity over the left dorsolateral prefrontal cortex (DLPFC) and subgenual anterior cingulate cortex (sgACC) as well as increased connectivity in the default (i.e. resting state) network in tinnitus patients with a maladaptive coping style. Correlation analysis revealed that the changes in the DLPFC correlate primarily with maladaptive coping behavior, whereas the changes in the sgACC correlate with tinnitus severity and depression. Our findings are in line with previous research in the field of depression that during resting state a alpha band hyperconnectivity exists within the default network for patients who use a maladaptive coping style, with the sgACC as the dysfunctional node and that the strength of the connectivity is related to focusing on negative mood and catastrophizing about the consequences of tinnitus.

## Introduction

It is well established chronic medical conditions can cause a high amount of burden in some patients, whereas other patients can tolerate the same medical condition much easier. Adaptively coping with chronic health impairment is a self-regulatory challenge. Failing to meet this challenge has serious consequences, as intrusive and emotionally charged thoughts about the disorder contribute decisively to the health related burden.

One chronic medical condition is tinnitus, a subjective auditory phantom phenomenon in which patients perceive an internal sound in the absence of an external sound source, which can be very distressing [1], [2]. Whereas more people who experience tinnitus can perfectly well live with it, 1 in 5 is emotionally affected by it [3], with 2.4% of the population suffering in the worst degree [4]. In those patients tinnitus is frequently accompanied by annoyance, concentration problems, depression, anxiety, irritability, sleep disturbances and intense worrying [5], [6]. Even if these symptoms have been traditionally considered as a learned reaction to tinnitus [1] their exact relationship to tinnitus is still a matter of debate [5]. Theoretically, it is also conceivable that these symptoms may be preceding the tinnitus onset and predispose for it or they may represent non-auditory symptoms resulting from the same pathophysiological changes that are involved in tinnitus generation [7]. One approach to answer this question is the investigation of the relationship between tinnitus related handicap and coping behavior, since coping is assumed to mediate the relationship between disease symptoms and the related handicap or distress [8], [9]. Previous research on tinnitus has already shown that the emotion regulation or coping style that tinnitus patients engage in is influencing tinnitus related stress [8], [9]. However, no study addressed the neural bases of successful and non-successful coping styles in tinnitus.

Functional imaging studies suggest that the subgenual anterior cingulate cortex (sgACC) plays an important role in adaptive versus maladaptive regulation of negative autobiographical memories [10]. The sgACC has been shown to be critically implicated in major depressive disorders (MDD) [11]–[14] and also in poor emotion regulation [15]. Recent structural and functional imaging studies suggest the involvement of the sgACC in tinnitus [16]–[18] and source localized EEG studies demonstrated an important role for this area in the amount of distress perceived by tinnitus patients [19], [20] and depressive feelings [21].

With respect to the neural bases of coping behavior it has been demonstrated that mind-wandering about the severity of a medical condition appears to engage regions of the default network, a set of brain areas that are active during rest periods, which include the posterior cingulate cortex, portions of lateral parietal cortex as well as the medial temporal lobe and medial prefrontal cortex [22]–[24]. It has recently been shown that MDD is characterized by increased default network connectivity and sgACC activity [25]. A seed-based connectivity approach further revealed that MDDs show more neural functional connectivity between the posterior cingulate cortex, which is part of the default network and the sgACC, than healthy individuals during rest periods [11].

In the present study we aimed to identify the neural bases of differences in coping behavior among tinnitus patients by using resting state electroencephalography (EEG). The study focuses on the cortical source differences in resting-state eyes closed EEG activity and connectivity between tinnitus patients who use different forms of coping. We differentiate between maladaptive, active and passive coping behavior. While maladaptive coping behavior involves focusing on negative mood, venting feelings and catastrophizing about the consequences of tinnitus, active coping behavior involves the use of a broad range of adaptive coping mechanisms. Active coping strategies are either behavioral or psychological responses designed to change the nature of the stressor itself or how one thinks about it. Passive coping behavior involves attempts to avoid tinnitus by masking the noises using background sounds and tinnitus maskers. It is further known that active styles are considered to be leading to improved psychosocial functioning, whilst maladaptive coping styles such as rumination about negative states are linked to increased depression. We hypothesize that a maladaptive coping style would be reflected in the default-mode network in tinnitus patients analogous to what is seen in depressed patients, generating increased activity of the sgACC and increased communication between the sgACC and the default network during resting state. We hypothesize that alpha activity might play an important role, as increased alpha activity has already been related to tinnitus related distress in previous EEG studies [19], [20].

## Methods

### Patients

Eighty-five tinnitus patients (57 males and 28 females) with a mean age of 48.20 years (Sd = 14.53 years) and a mean tinnitus duration of 6.02 years (Sd = 7.54 years) were selected from the multidisciplinary Tinnitus Research Initiative (TRI) Clinic of the University Hospital of Antwerp, Belgium. For the clinical and demographic characterization of the sample see Table 1. Individuals with pulsatile tinnitus, Ménière disease, otosclerosis, chronic headache, neurological disorders such as brain tumors, and individuals being treated for mental disorders were not included in the study in order to obtain a homogeneous sample. All patients were investigated for the extent of hearing loss using audiograms. Tinnitus matching was performed looking for tinnitus pitch (frequency) and tinnitus intensity.

**Table 1:** Population statistics.

		Coping style		Total
		adaptive	maladapative	
Gender	Male	35	22	57
	Female	14	14	28
				
Age	*Mean*	49.04	47.06	48.20
	Sd	13.56	14.78	14.53
				
Tinnitus type	Pure tone	22	15	37
	Narrow Band Noise	27	21	48
				
Tinnitus lateralization	Unilateral	24	16	40
	Bilateral	25	20	45
				
Tinnitus Duration	*Mean*	5.78	6.34	6.02
	Sd	5.79	9.24	7.54
				
Hearing loss	*Mean*	27.18	32.05	29.24
(dB HL)	*SD*	14.59	16.43	15.03

Antwerp University Ethics Committee reviewed and approved the study and all applicable documents prior to study initiation. All patients signed an approved informed consent in order to enroll into the study.

### Questionnaires

#### TCSQ

A Dutch translation was made from the Tinnitus Coping Style Questionnaire (TCSQ). This questionnaire assesses the frequency in which sufferers use a broad range of tinnitus-specific coping actions to manage the intrusiveness of the tinnitus sound. The questionnaire identifies three tinnitus coping behaviors, namely maladaptive coping, effective or active coping, and passive coping [8], [26]. One item was excluded from the questionnaire (‘Using a pillow speaker to help you sleep’) as most tinnitus patients are not familiar with this product. As such, the translated TCSQ consisted of 39 items measured on a 7-point scale, ranging from ‘never’ (scored as 1) to ‘always’ (scored as 7). This questionnaire was used as the primary outcome measure as this questionnaire was specifically developed to measure coping behavior in tinnitus.

To validate the TCSQ in Dutch the COPE, Beck Depression Scale (BDI), the Tinnitus Questionnaire (TQ), Visual Analogue Scale (VAS) for tinnitus loudness were measured.

#### COPE

A Dutch translation of the COPE [27] was used, a coping inventory that includes 53 items and exist out of 13 subscales: Active coping, Planning, Suppression of competing activities, Restraint coping, Seeking social support for instrumental reasons, Seeking social support for emotional reason, Positive reinterpretation & growths, Acceptance, Turning to Religion, Focus on & venting of emotions, Denial, Behavioral disengagement, Mental disengagement, Alcohol-drug disengagement [28].

#### VAS

A visual analogue scale for loudness (‘How loud is your tinnitus?’) was assessed.

#### TQ

We used the Dutch translation of the Tinnitus Questionnaire validated by Meeus et al. [29]. This scale comprised of 52 items and is a well-established measure for the assessment of a broad spectrum of tinnitus-related psychological complaints. The TQ measures emotional and cognitive distress, intrusiveness, auditory perceptual difficulties, sleep disturbances, and somatic complaints. As previously mentioned, the global TQ score can be computed to measure the general level of psychological and psychosomatic distress. A 3-point scale is given for all items, ranging from ‘true’ (2 points) to ‘partly true’ (1 point) and ‘not true’ (0 points). The total score (from 0–84) was computed according to standard criteria published in previous work [29], [30].

#### BDI

Beck Depression Inventory is a depression test to measure the severity and depth of depression symptoms. Each of the inventory items corresponds to a specific category of depressive symptom and/or attitude according to DSM-IV. This questionnaire was validated in Dutch [31].

### EEG data collection

EEG recordings (Mitsar-201, NovaTech http://www.novatecheeg.com/) were obtained in a quiet and dimly lighted room with each participant sitting upright on a small but comfortable chair. Participants were requested to abstain from alcohol consumption 24 hours prior to recording, and from caffeinated beverages consumption on the day of recording. The actual recording lasted approximately 5 min. The EEG was sampled with 19 electrodes in the standard 10–20 International placement referenced to linked ears and impedances were checked to remain below 5 kΩ. Data were collected eyes-closed (sampling rate = 1024 Hz, band passed 0.15–200 Hz). Data were resampled to 128 Hz, band-pass filtered (fast Fourier transform filter) to 2–44 Hz and subsequently transposed into Eureka! Software [32], plotted and carefully inspected for manual artifact-rejection (i.e. eye blinks, eye movements, teeth clenching, body movement, or ECG artifact) and removed. Average Fourier cross-spectral matrices were computed for bands delta (2–3.5 Hz), theta (4–7.5 Hz), alpha1 (8–10 Hz), alpha2 (10–12 Hz), beta1 (13–18 Hz), beta2 (18.5–21 Hz), beta3 (21.5–30 Hz) and gamma (30.5–44 Hz).

### Source localization

Standardized low-resolution brain electromagnetic tomography (sLORETA) was used to estimate the intracerebral electrical sources that generated the scalp-recorded activity in each of the eight frequency bands [33]. sLORETA computes electric neuronal activity as current density (A/m^^2^^) without assuming a predefined number of active sources. The sLORETA solution space consists of 6,239 voxels (voxel size: 5×5×5 mm), computations were made in a realistic head model, and is restricted to cortical gray matter and hippocampi, as defined by digitized MNI152 template [34]. Scalp electrode coordinates on the MNI brain are derived from the international 5% system [35].

### Lagged phase coherence (connectivity)

Lagged phase coherence between two sources can be interpreted as the amount of cross-talk between the regions contributing to the source activity [36]. Since the two brain areas oscillate coherently with a phase lag, the cross-talk can be interpreted as information sharing by axonal transmission. More precisely, the discrete Fourier transform decomposes the signal in a finite series of cosine and sine waves (in-phase and out-of-phase carrier waves, forming the real and imaginary part of the Fourier decomposition) at the Fourier frequencies. The lag of the cosine waves with respect to their sine counterparts is inversely proportional to their frequency and amounts to a quarter of the period; The threshold of significance for a given lagged phase coherence value according to asymptotic results can be found as described by Pascual-Marqui [37], [38], where the definition of lagged phase coherence can be found as well.

Time-series of current density were extracted for different regions of interest using sLORETA. Power in all 6,239 voxels was normalized to a power of 1 and log transformed at each time point. Region of interest values thus reflect the log transformed fraction of total power across all voxels, separately for specific frequencies. Regions of interest were defined based on previous brain research on default network by Fox et al. [39] as well as the findings based on source localization (BA25, BA9/46) on tinnitus (Table 2).

**Table 2:** Default network [39].

Common names	Brodmann's areas
Posterior cingulate cortex	BA 31
Retro-splenial cortex	BA 30
Lateral parietal cortex	BA 39
Medial prefrontal cortex	BA 32/10
Superior frontal cortex	BA 8
Inferior temporal cortex	BA 20/21
Parahippocampal gyrus	BA 35

### Region of interest analysis

The log-transformed electric current density was averaged across all voxels belonging to the region of interest, respectively BA25 left and right, BA9/46 left and BA9/46 right separately across all frequency bands.

### Statistical analyses

The methodology used is non-parametric. It is based on estimating, via randomization, the empirical probability distribution for the max-statistic, under the null hypothesis comparisons [40]. This methodology corrects for multiple testing (i.e., for the collection of tests performed for all voxels and for all frequency bands). Due to the non-parametric nature of the method, its validity does not rely on any assumption of Gaussianity [40]. Statistical contrast maps were calculated through multiple voxel-by-voxel comparisons in a logarithm of F-ratio. The significance threshold was based on a permutation test with 5000 permutations. A comparison was made between tinnitus patients using an adaptive coping style in comparison to tinnitus patients using a maladaptive coping style as well as correlation were calculated with different questionnaires.

Factor analysis using principal component extraction was performed on the TCSQ. Based on the scree test plot the number of components is determined. Internal consistency for the different components was calculated using Cronbach Alpha. To verify the construct validity the components were correlated with different subscales of the COPE questionnaire using Pearson correlations. To further verify the convergent validity the different components of the TCSQ were correlated with the BDI, the subscales of the TQ, the total score of the TQ, and the VAS loudness.

In order to differentiate the sample in groups, a hierarchical cluster analysis based on the squared Euclidean distance was conducted on the 39 coping items of the TCSQ. A multivariate ANOVA with the 3 components as dependent variables and the cluster groups as an independent variable was conducted to further explore these different clusters. In addition, a multivariate analysis was conducted with the clusters as independent variable and VAS loudness, BDI, and the TQ subscales (emotional aspects, cognitive aspects, intrusiveness, perceptual differences, sleep disturbance, and somatic problems) as dependent variables.

To compare sample correlation coefficients drawn from the same sample we rely on a formula described in Cohen and Cohen [41]. The formula yields in a t-statistic with n-3 degrees of freedom. The formula tests for a significant difference in the correlation between variables X & Y and V & Y.

## Results

### Coping behavior and coping style

**Figure 1:**
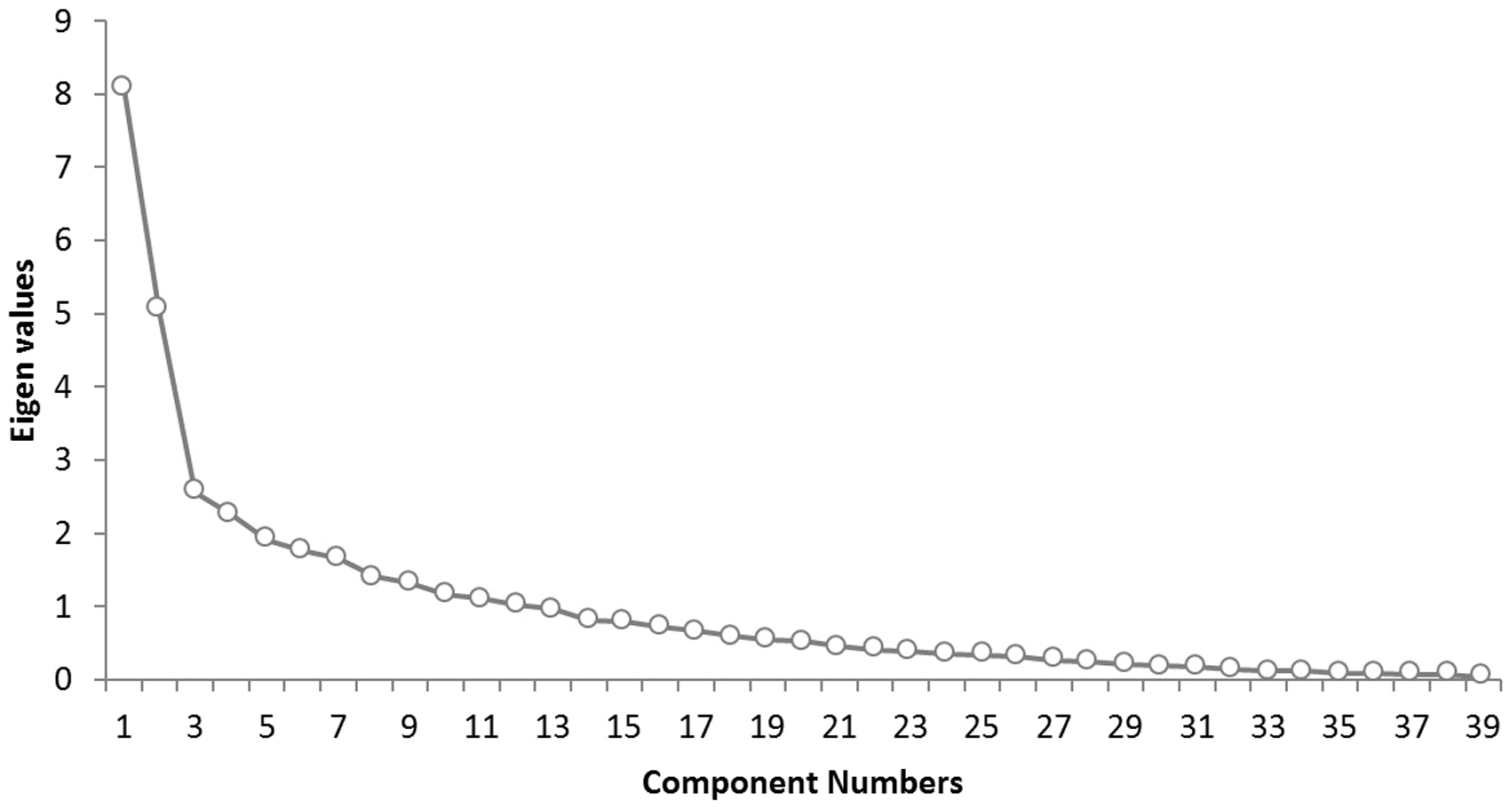
Scree plot for the THQ shows the number of components and the corresponding eigenvalues (see Figure 1). When the drop ceases and the curve makes an elbow toward less steep decline, all further components after the one starting the elbow can be dropped. The scree plot indicates that three factors would be ideal.

Factor analysis using principal component extraction was performed on the TCSQ. A scree plot indicates that three factors would be ideal (Figure 1). The first factor explained 20.75% of the total variance (eigenvalue = 8.09), a second factor explained 12.95% of the total variance (eigenvalue = 5.05) and a third factor 6.58% of the total variance (eigenvalue = 2.57). Table 3 presents the factor pattern matrix after oblique rotation. Similar to previous research using the same questionnaire [8], [26], factor 1 corroborates with *maladaptive coping behavior*, factor 2 with *active coping behavior*, and factor 3 with *passive coping behavior*. These three factors show a good internal consistency measured with Cronbach α coefficient: maladaptive coping behavior (α = .88), active coping behavior (α = .76), and passive coping behavior (α = .69).

**Table 3:** The items for the TCSQ. Results from the factor analysis using principal component extraction.

	Component		
	Factor 1	Factor 2	Factor 3
	maladaptive	active	passive
1. Ik gebruik bepaalde middelen om mijn tinnitus te maskeren.	.12	.00	**.49**
*Using a masker for tinnitus*			
2. Ik probeer aan plezante dingen te denken, in plaats van me op mijn tinnitus te concentreren.	.09	**.71**	.06
*Thinking of something pleasant rather than concentrating on your tinnitus*			
3. Ik denk erover om het op te geven.	**.44**	−.13	.29
*Thinking of giving up*			
4. Ik probeer mezelf eraan te herinneren dat mijn leven over het algemeen voldoening geeft en bevredigend is.	−.08	**.57**	.00
*Reminding yourself that your life is generally fulfilling and satisfying*			
5. Nadenken hoe erg en onplezierig dit geluid is.	**.70**	.11	−.03
*Thinking about how awful and unpleasant the noises sound*			
6. Gebruik maken van achtergrondlawaai om te helpen slapen.	.02	.04	**.74**
*Using background noise mask your tinnitus*			
7. Bidden helpt om mijn tinnitus te doen verminderen of te laten verdwijnen.	.13	.08	**.32**
*Praying yours tinnitus will suddenly diminish or stop*			
8. Ik doe bewust moeite om mijn tinnitus weg te denken.	.30	**.60**	.11
*Making a conscious effort to think your tinnitus away*			
9. Ik vertel andere hoe erg mijn tinnitus is.	**.79**	.17	−.12
*Telling others how awful your tinnitus is*			
10. Ik dagdroom over hoe mijn leven zou zijn zonder tinnitus.	**.78**	.15	.14
*Daydreaming about what life would be like without tinnitus*			
11. Ik vraag mij af waaraan ik mijn tinnitus verdiend heb.	**.68**	.14	.17
*Asking what you have done to deserve your tinnitus*			
12. Luisteren naar de radio, muziek of TV kijken maskeert mijn tinnitus.	−.14	.26	**.59**
*Listening to the radio, music, or watching TV to mask your tinnitus*			
13. Ik bekijk tinnitus als een deel van het alledaagse achtergrond lawaai.	−.19	**.64**	.05
*Thinking of your tinnitus as part of everyday background noise*			
14. Ik doe alsof mijn tinnitus er niet is.	−.24	**.58**	.22
*Pretending your tinnitus is not there*			
15. Ik vermijd sociale situaties als gevolg van mijn tinnitus.	**.56**	−.12	.00
*Avoiding social situations because of your tinnitus*			
16. Ik focus mij volledig op de dingen waar ik mee bezig ben, of op dingen die gebeuren rondom mij.	−.04	**.38**	−.15
*Focusing your attention fully on what you are doing, or on the things that are happening around you*			
17. Ik kruip in mijn bed en/of slaap tijdens de dag om van mijn tinnitus af te zijn.	.28	−.27	**.35**
*Going to bed and/or sleeping during the day to get away from your tinnitus*			
18. Ik verzeker mezelf ervan dat ik mijn tinnitus kan tolereren/negeren.	**−.45**	.30	.12
*Reassuring yourself that you can learn to tolerate/ignore your tinnitus*			
19. Ik probeer niet aan mijn tinnitus te denken.	−.39	**.59**	.17
*Trying not to think about your tinnitus*			
20. Ik consulteer een therapeut of psycholoog om nieuwe manieren te leren om met mijn tinnitus om te gaan.	.03	−.18	**.64**
*Consulting a professional counselor, or psychologist, to learn new ways of coping with tinnitus*			
21. Ik neem hobby's en passies op om mezelf af te leiden van mijn tinnitus.	.27	**.36**	.28
*Taking up hobbies and interests to distract yourself from your tinnitus*			
22. Ik denk dat tinnitus mijn levenskwaliteit heeft geruïneerd.	**.72**	−.19	.30
*Thinking that your tinnitus has ruined the quality of your life*			
23. Ik verzeker mezelf ervan dat ik kan omgaan met mijn tinnitus, aangezien dat ik in het verleden ook heb gedaan.	**−.53**	.18	.38
*Reassuring yourself that you can cope with your tinnitus now because you have coped in the past*			
24. Ik luister vaak naar mijn tinnitus.	**.68**	−.01	−.03
*Listening to your tinnitus*			
25. Ik denk aan dingen om te doen om mijzelf van mij tinnitus af te leiden.	.31	**.59**	.02
*Thinking of things to do to distract yourself from your tinnitus*			
26. Ik probeer mij eraan te herinneren dat ik nog altijd van het leven kan genieten.	−.03	**.73**	−.06
*Reminding yourself that you can still enjoy life despite your tinnitus*			
27. Ik hoop dat er binnenkort een oplossing kan gevonden worden voor tinnitus.	**.32**	.22	−.02
*Hoping that a cure for tinnitus will be found soon*			
28. Ik zeg tegen mijzelf dat tinnitus gewoon één van de uitdagingen in het leven is.	−.35	**.50**	.19
*Saying to yourself that tinnitus is just one of life's many challenges*			
29. Ik neem voorgeschreven medicatie om mij te helpen slapen.	**.40**	−.19	.30
*Taking prescribed medication to help your tinnitus*			
30. Ik kijk naar mensen rondom mij die in een ergere situatie zitten dan ik.	**.37**	.27	.04
*Looking at others around you who are in a worse situation than yourself*			
31. Ik denk vaak aan vroegere tijden waar ik geen tinnitus had.	**.67**	−.01	.07
*Thinking of times in the past when you did not have tinnitus*			
32. Ik probeer bezig te blijven of actief te zijn om mijzelf af te leiden van mijn tinnitus.	.34	**.50**	−.14
*Staying busy or active to distract yourself from your tinnitus*			
33. Ik denk dat ik niets kan doen om te leren omgaan met mijn tinnitus.	**.51**	−.28	.25
*Thinking that you cannot do anything to cope with your tinnitus*			
34. Ik laat tinnitus niet mijn leven beheersen.	**−.39**	.06	.26
*Thinking that you won't let your tinnitus get the better of you*			
35. Ik verzeker mezelf ervan dat ik toegang heb tot professioneel advies en ondersteuning.	.11	−.07	**.30**
*Reassuring yourself that you have access to professional advice and support*			
36. Ik lees om mij af te leiden van mijn tinnitus.	.06	**.33**	−.02
*Reading in order to distract yourself from your tinnitus*			
37. Ik ben bang dat het geluid mij een zenuwinzinking zal bezorgen.	**.54**	−.05	.32
*Worrying that the noises will give you a nervous breakdown*			
38. Ik leer en gebruik relaxtatie technieken	−.25	.21	**.52**
*Learning and practicing relaxation techniques*			
39. Ik denk dat ik niet instaat ben om, om te gaan met mijn tinnitus.	**.70**	−.18	.27
*Thinking about not being able to put up with tinnitus*			

	Correlation		
	Factor 1	Factor 2	Factor 3
	maladaptive	active	passive
**Factor 1** maldaptive	-	−.07	.16
**Factor 2** active	-	-	.08
**Factor 3** passive	-	-	-

The communality is the sum of the squared correlations between a variable and each of the three factors.

Extraction Method: Principal Component Analysis.

Rotation Method: Oblique with Kaiser Normalization.

a. Rotation converged in 7 iterations.

To verify the construct validity of the TCSQ we correlated the different subscales with the 13 subscales of COPE. Correlation analyses revealed for *maladaptive coping behavior as measured by the TCSQ* a positive correlation with the following items of COPE: *suppression of competing activities*, *seeking social support for emotional reasons*, *focus on and venting of emotions*, *behavioral disengagement*, *mental disengagement* and a negative correlation with *positive reinterpretation and growth* (Table 4). The subscale *active coping behavior* of the TCSQ correlated positively with *active coping*, *positive reinterpretation and growth*, and *mental disengagement of the COPE questionnaire*. The TCSQ subscale *passive coping behavior* correlated positively with COPE's *restraint coping*, and *focus on and venting of emotions*.

**Table 4:** Correlation analysis between the TCSQ and respectively COPE, Tinnitus loudness, Tinnitus distress, BDI, the different subscales of the TQ and the total score of the TQ.

	TCSQ Coping behavior		
	maladaptive	active	passive
***COPE scale***			
*Active coping*	.18	.31**	.07
*Planning*	.20†	−.08	.19†
*Suppression of competing activities*	.29**	−.14	.15
*Restraint coping*	.14	−.05	.21*
*Seeking social support for instrumental reasons*	.14	−.08	.00
*Seeking social support for emotional reasons*	.23*	.03	.12
*Positive reinterpretation & growth*	−.50***	.24*	.00
*Acceptance*	−.59***	.24*	−.08
*Turning to Religion*	.13	.20	−.07
*Focus on & venting of emotions*	.54***	.01	.30**
*Denial*	.17	.02	.02
*Behavioral disengagement*	.40**	−.03	.05
*Mental disengagement*	.28**	.33**	.15
*Alcohol-drug disengagement*	.07	.11	.03
**Tinnitus loudness**	.38***	.01	.04
**BDI**	.65***	.00	.21
***TQ***			
*distress (emotional)*	.59***	−.02	.21
*distress (cognitive)*	.68***	−.05	.00
*intruisiveness*	.66***	−.16	.01
*perceptual difficulties*	.45***	−.15	−.05
*sleep distubance*	.41***	.07	.05
*somatic problems*	.46***	.12	.11
*total*	.77***	−.05	.09

† *p*<.10;

* *p*<.05;

** *p*<.01;

*** *p*<.001.

Convergent validity was found by correlating the TCSQ with typical measures used in tinnitus research indicating that tinnitus loudness, tinnitus distress, BDI, the different subscales of the TQ and the total score of the TQ correlated positively with maladaptive coping behavior, but not with active and passive coping (Table 4).

In order to differentiate the sample in groups that differ in their general coping behavior a hierarchical cluster analysis based on the squared Euclidean distance was conducted on the 39 coping items. This analysis revealed that 2 clusters (i.e. 2 groups) give a reliable solution. A multivariate ANOVA with the 3 TCSQ coping dimensions as dependent variables and the 2 cluster groups as an independent variable indicates a significant effect, *F* = 61.32, *p*<.001. Univariate analysis indicates that the effect can mainly be explained by a significant effect of factor 1 *maladaptive coping behavior*, but not by factor 2 *active coping behavior* and factor 3 *passive coping behavior* of the TCSQ (Table 5). For maladaptive behavior it was shown that the second cluster had a higher score on this dimension in comparison to the first cluster. As such, one can interpret cluster 1 as patients making use of adaptive coping styles, while the second group uses maladaptive coping styles.

**Table 5:** A comparison of the mean score on the three factors for the two cluster groups.

	Coping style		
	adaptive	maladaptive	*F*-value
TCSQ maladaptive coping behavior	2.72	4.39	188.37***
TCSQ active coping behavior	3.96	3.74	1.52
TCSQ passive coping behavior	2.64	2.95	1.40

* *p*<.05;

** *p*<.01;

*** *p*<.001.

A multivariate analysis with groups *adaptive coping style* versus *maladaptive coping style* as independent variable and VAS loudness, BDI and the TQ subscales (emotional aspects, cognitive aspects, intrusiveness, perceptual differences, sleep disturbance and somatic problems) as dependent variables revealed a significant effect, *F* = 8.42, *p*<.001. Univariate analysis revealed that the maladaptive group has higher scores on the different dependent variables in comparison to the adaptive group (Table 6).

**Table 6:** Tinnitus loudness, Tinnitus annoyance, BDI, subscale of TQ (emotional distress, cognitive distress, intrusiveness, perceptual differences, sleep disturbance, somatic problems) & Total score on the TQ.

	Coping style		
	adaptive	maladaptive	
Tinnitus loudness	5.21	6.60	23.35***
Tinnitus annoyance	4.06	6.91	5.11*
BDI	7.20	19.20	39.07***
*TQ*			
*distress (emotional)*	4.91	11.57	55.34***
*distress (cognitive)*	5.13	8.05	28.04***
*intruisiveness*	9.88	13.66	31.51***
*perceptual difficulties*	4.56	6.98	17.79***
*sleep distubance*	2.28	4.18	5.14*
*somatic problems*	2.72	4.77	15.87**
Total	29.47	49.20	8.42***

* *p*<.05;

** *p*<.01;

*** *p*<.001.

### Neural correlates of the 2 clusters: maladaptive vs. adaptive coping styles

**Figure 2:**
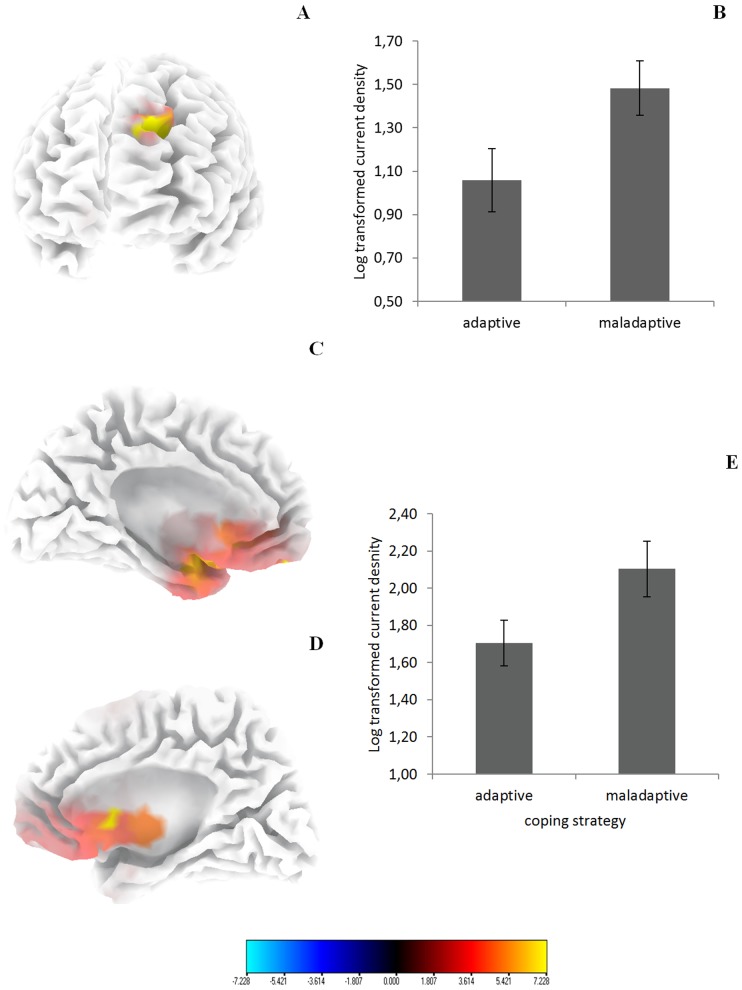
(A) Increased activity in the left dorsolateral prefrontal cortex (BA9) for tinnitus patients using a maladaptive coping style in comparison to tinnitus patients using an adaptive coping style for the frequency band Alpha1. (B) Region of interest analysis shows increased activity in the left dorsolateral prefrontal cortex (BA9) for tinnitus patients using a maladaptive coping style in comparison to tinnitus patients using an adaptive coping style for Alpha1. (C & D) Increased activity in the subgenual anterior cingulate cortex (BA25) for tinnitus patients using a maladaptive coping style in comparison to tinnitus patients using an adaptive coping style for the frequency band Alpha2. (E) Region of interest analysis shows increased activity in the subgenual anterior cingulate cortex (BA25) for tinnitus patients using a maladaptive coping style in comparison to tinnitus patients using an adaptive coping style for Alpha2.

For the alpha 1 and alpha2 band sLORETA revealed a higher current source density for the maladaptive coping style as compared to the adaptive coping style over the left dorsolateral prefrontal cortex (DLFPC) (Figure 2A) and sgACC (BA25) (Figure 2C&D). In the other frequency bands (delta, theta, beta 1, beta 2, beta 3 and gamma) there were no statistically significant differences between the two groups.

To confirm these results and based on previous research a ROI analysis of these two regions was conducted. An ANOVA with coping style (maladaptive versus adaptive coping) as independent variable and log-transformed current density for respectively the left DLPFC and sgACC revealed a significant effect for coping style, demonstrating that the maladaptive coping style had a higher log-transformed current density in comparison to the adaptive coping style for respectively the left DLPFC (*F* = 4.83, *p*<.05; Figure 2B) and sgACC (*F* = 4.30, *p*<.05; Figure 2E).

### Maladaptive coping behavior and the left dorsolateral prefrontal cortex

**Figure 3:**
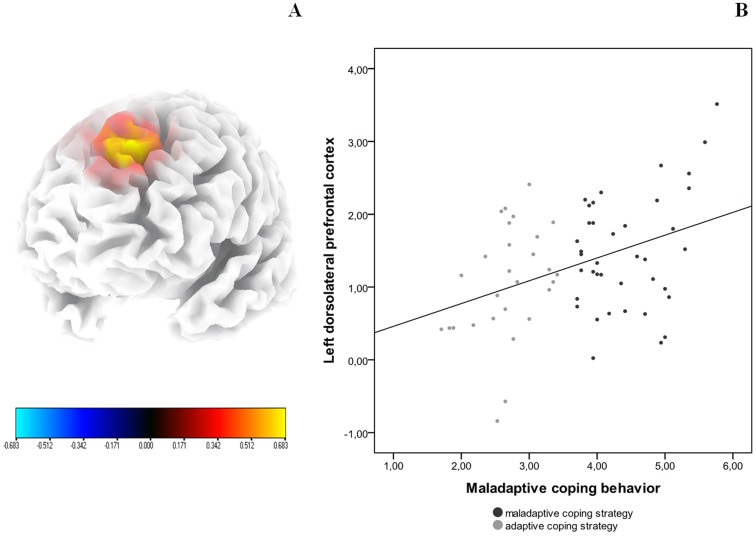
(A) A significant positive correlation between maladaptive coping behavior and alpha1 activity in the left dorsolateral prefrontal cortex on whole brain analysis. (B) A significant positive correlation after a ROI analysis between the left DLPFC (BA9/46) and maladaptive coping behavior.

A correlation analysis on the whole brain with maladaptive coping behavior indicates a significant positive correlation between the left dorsolateral prefrontal cortex (DLPFC) and maladaptive coping behavior for the alpha 1 band (Figure 3A,B). No significant effects were obtained for delta, theta, alpha2, beta 1, beta 2, beta 3 and gamma frequency band. Similar analyses with respectively the active coping behavior and passive coping behavior yielded no significant effects.

To further confirm this finding and based on previous research, a ROI analysis was conducted correlating respectively the left and right DLPFC with the maladaptive coping behavior. This analysis revealed a significant positive correlation between the left DLPFC and maladaptive coping behavior (r = .40, *p*<.001; Figure 3B), but not with the right DLPFC (r = .17, *p* = .14). To further explore this latter effect, alpha 1 activity within the left DLPFC was also correlated with respectively the BDI (r = .27, *p*<.05), TQ (r = .14, n.s.), and VAS loudness. To compare sample correlation coefficients between the left DLPFC and respectively the maladaptive coping behavior and BDI an additional analysis was conducted revealing that the correlation was marginally significantly; stronger for maladaptive coping behavior than for the BDI (t = 1.37, *p* = .09). A similar analysis for active coping behavior and passive coping behavior groups did not yield in significant effects.

### Maladaptive coping behavior and the subgenual Anterior Cingulate Cortex

To further evaluate the involvement of the sgACC in *maladaptive coping behavior, TQ, BDI, and VAS loudness*, a region of interest of the sgACC was extracted for alpha2 activity and correlated to each of these characteristics (maladaptive coping behavior, TQ, BDI, VAS loudness). A significant correlation was found for sgACC alpha2 activity and the TQ (r = .34, *p*<.01) as well as the BDI (r = .24, *p*<.05), but not for maladaptive coping behavior (r = .08, *n.s.*) and VAS loudness (r = −.08, *n.s.*).

### Differences within the default network between tinnitus patients groups with maladaptive vs. adaptive coping styles

**Figure 4:**
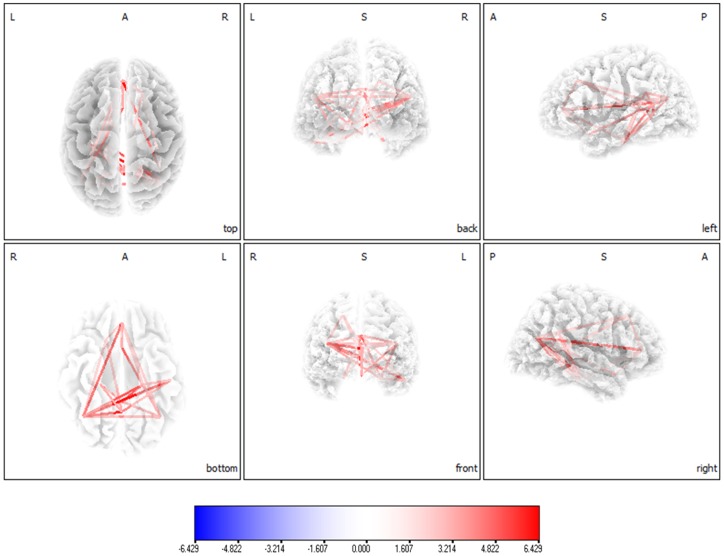
Connectivity analysis (lagged phase synchronization) yielded in a significant difference (*p*<.05) between maladaptive and adaptive tinnitus patients for the alpha2 frequency band. Increased lagged phase coherence could be found in general for tinnitus patients using a maladaptive coping style in comparison to tinnitus patients using an adaptive coping style in default network extending to the sgACC.

Connectivity analysis yielded in a significant difference (*p*<.05) between maladaptive and adaptive tinnitus patients for the alpha2 frequency band (Figure 4). Increased lagged phase coherence could be found in general for tinnitus patients using a maladaptive coping style in comparison to tinnitus patients using an adaptive coping style in default network extending to the sgACC. No significant effects were obtained for delta, theta, alpha1, beta1, beta2, beta3 and gamma.

### Correlation analysis: Default network of maladaptive coping behavior

**Figure 5:**
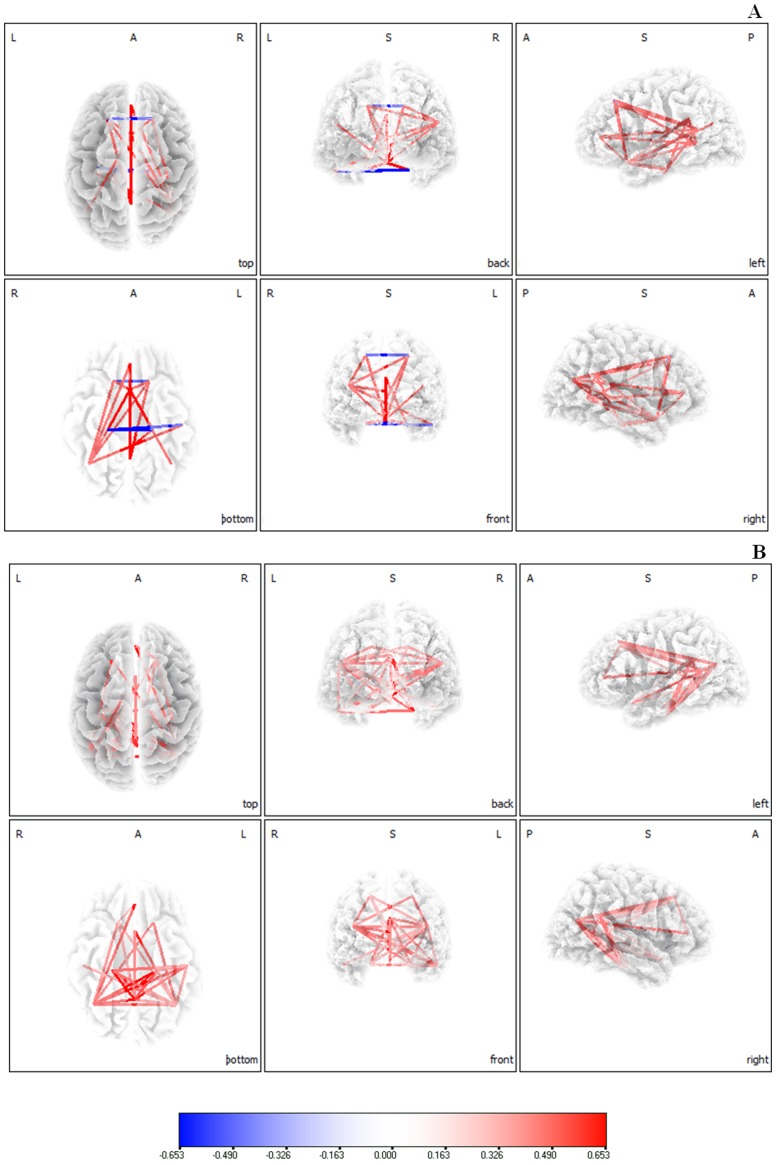
A correlation analysis between lagged phase coherence and maladaptive coping behavior revealed significant effects for the alpha 1 and alpha2 default network. (A) Increased functional connectivity (lagged phase synchronization) correlated with maladaptive behavior for the default network in Alpha1. (B) Increased functional connectivity (lagged phase synchronization) correlated with maladaptive behavior for the default network in Alpha2.

A correlation analysis between lagged phase coherence and maladaptive coping behavior revealed significant effects for the alpha 1 and alpha2 default network (Figure 5 A & B) which also includes the sgACC. No significant effects were obtained for the other frequency bands (delta, theta, beta1, beta2, beta3 and gamma). Similar analyses were conducted between lagged phase coherence and BDI, TQ, VAS loudness, Age for alpha 1 and alpha2. These additional analyses revealed no significant effects.

## Discussion

In the present study, we examined cortical differences in resting-state EEG activity between tinnitus patients who use an adaptive coping style versus those who use a maladaptive coping style.

### Coping behavior and style

We demonstrated that the Tinnitus Coping Style Questionnaire is a reliable and valid questionnaire measuring coping behavior on three dimensions, namely *maladaptive*, *active* and *passive* coping behavior. These three factors are similar to previous research on tinnitus coping [8] or coping with general life stress [42]. Furthermore, we found that tinnitus loudness, tinnitus distress, BDI, the different subscales of the TQ, and the total score of the TQ correlated positively with maladaptive coping behavior, but not with active and passive coping. This finding is in line with earlier studies investigating the relationship between TCSQ results and tinnitus related handicap [26].

Based on these three coping behaviors a cluster analysis indicated that some tinnitus patients apply an adaptive coping style, while others apply a maladaptive coping style. The latter group had higher scores on maladaptive coping behavior, but not on active and passive coping behavior. The tinnitus patients using a maladaptive coping style are characterized by increased scores on the depression scale, tinnitus questionnaire, and experienced their tinnitus as louder and more distressing in comparison to tinnitus patients using an adaptive coping style. This fits with previous results that showed that a maladaptive coping style is linked with increased depression [43]. It is possible that tinnitus patients suffering from depression are not able to use adaptive coping styles or, vice versa, the inability to use adaptive coping could lead to depression. It is known that depressive patients use maladaptive coping styles more frequently in comparison to healthy subjects [44]. These encompass rumination about negative affect, catastrophizing and self-blame in response to negative events. However, further longitudinal research is needed to have a better understanding on how coping styles relate to coping with tinnitus and its relationship with depression.

### Coping behavior, style, and the neural correlates

Activity changes were obtained in the left DLPFC and sgACC alpha 1 and alpha 2 frequency band. Our results indicated increased activity when patients applied a maladaptive coping strategy in comparison to adaptive coping strategy. A positive association was found between increased alpha1 activity in the left DLPFC with maladaptive coping behavior and a positive association was found between alpha2 activity in the sgACC and tinnitus related distress and depressive feelings. Furthermore increased lagged phased synchronization was noted between the default network and sgACC for the alpha2 frequency band for patients using a maladaptive coping style, and the higher the lagged phased synchronization was between different parts of the default network and the sgACC the higher the tinnitus patients scored on maladaptive coping behavior for respectively alpha1 and alpha2 frequency bands.

The involvement of the DLPFC and sgACC in tinnitus is not that rare. Both areas are already discussed in previous studies on tinnitus [19], [21]. It is known that the DLPFC plays an important role in anxiety [45], the unpleasantness related to pain [46] and in aversive auditory stimuli [47]. Further support for the involvement of the DLPFC in tinnitus stems from studies using brain stimulation. High frequency rTMS and tDCS of the left DLPFC is capable of reducing tinnitus severity [48]–[54] as well as enhancing effects of temporal cortex rTMS [55]. In addition, previous research has demonstrated that the distress network in tinnitus is characterized by increased alpha activity in the sgACC [16], [19], [20]. Highly distressed tinnitus patients have increased alpha activity within the sgACC in comparison to tinnitus patients with low distress [16], [19], [20]. Furthermore there is an increased functional connectivity between the parahippocampal area and the sgACC at 10 Hz and 11.5 Hz in grade III and grade IV distress respectively [16]. The sgACC has gained considerable attention both for its putative role in a mood-regulation circuit and for its specific role in depression [25], [56], pain distress [57], [58], distress in asthmatic dyspnea [59], [60], and distress in functional somatic syndromes such as electro-sensitivity for mobile phones [61], social rejection distress [10], as well as tinnitus [19], [62]. In the depression literature activation of the sgACC has been associated with autonomic responses of emotional processing during rumination and brooding [11]. Our findings are in agreement with this previous research and add the important role of these brain areas in tinnitus specifically when patients apply a maladaptive coping style.

Our findings indicate that increased maladaptive coping behavior is associated with an increased resting state functional connectivity in the default network in tinnitus patients and is connected to sgACC. The sgACC is considered an important dysfunctional node in MDD [25], [56], [63] and associated with functional connectivity increases with increasing length of depressive episode. A ‘hyper’-connectivity in the default system extending to the sgACC during resting state in MDD has been associated to depressive rumination [25], [56], [63] or rumination during idle moments [11]. This corroborates with our findings and suggests that the resting-state signal in the sgACC region may be a marker for refractoriness to treatment [25]. The involvement of the sgACC is in agreement with the recent tinnitus model introduced by Rauschecker et al. [62] suggesting that the sgACC/ventromedial prefrontal cortex might be a central hub in tinnitus and that this limbic structure shows a stronger hyperactivity and structural differences in tinnitus patients than the auditory cortex in comparison to control subjects [17]. It is known that subgenual region is extremely rich in serotonin transporters and is considered as a governor for a vast network. Rauschecker and colleagues [62] reinvoked the serotonin hypothesis that suggested that tinnitus goes together with a serotonin depletion [64], [65] in addition to hypersensitivity to noise reduced REM sleep and depression [66], [67]. Interestingly, recent research revealed that serotonin depletion in animals within the medial prefrontal cortex induces stress and passive coping [68]. Furthermore the default mode network is involved in self-referential processing [69], mindwandering [70], thinking about the future [71] and the past [72], as well as in goal directed cognition, coping and goal directed behavior [73]. When the sgACC, which is involved in tinnitus loudness and distress processing [16], [17], [19]–[21], [62], becomes tightly coupled to this default network, the tinnitus becomes coupled to the mind-wandering and self-referential processing resulting in tinnitus related ruminating [74]. This coupling potentially also explains why maladaptive coopers perceive their tinnitus as louder as the noise cancelling mechanism is possibly inhibited by alpha activity, which can have an inhibitory function in the auditory system [75].

### The role of alpha band

The electrophysiological correlate of self-referential processing in the default mode network is alpha activity [76]–[78], and alpha activity correlates with fMRI BOLD activity in the default mode network [79], [80]. During periods when attention is focused internally (mind wandering) there is more neural phase synchronization between brain regions associated with the default network, whereas during periods when subjects are focused on performing a more neural phase synchrony within a task-specific brain network is noted [81]. As in maladaptive coping people are constantly ruminating about their tinnitus, it seems logical that their default mode system is hyper-connected in the default mode's physiological frequency and that the more they ruminate the more alpha synchronization they have in the DMN. If this is corrected it can be predicted that in a study that looks at the percentage of the time that patients focus on their tinnitus in a day will be correlated with alpha synchronization within the DMN and between the DMN and the sgACC. We need to conduct further research to explore this latter proposition.

## Conclusion

In summary, the present findings add to prior work suggesting that the DLPFC and sgACC are involved in tinnitus patients who use a maladaptive coping style leading to the probability to display more distressed and depressed behavior. Our results extend previous research in the field of depression by demonstrating that maladaptive coping style is related to alpha “hyper”-connectivity within the default network during rest as well as with the sgACC. The strength of the connectivity is related to focusing on negative mood and catastrophizing about the consequences of tinnitus (i.e. maladaptive coping behavior).
